# Endogenous physical regulation of population density in the freshwater protozoan *Paramecium caudatum*

**DOI:** 10.1038/s41598-017-14231-0

**Published:** 2017-10-23

**Authors:** Daniel Fels

**Affiliations:** University of Basel, Dept. of Environmental Sciences, Hebelstrasse 1, 4056 Basel, Switzerland

## Abstract

Studies confirm physical long-range cell-cell communication, most evidently based on electromagnetic fields. Effects concern induction or inhibition of cell growth. Their natural function is unclear. With the protozoan *Paramecium caudatum* I tested whether the signals regulate cell density and are electromagnetic. Up to 300 cells/mL, cell growth in clones of this study is decreasingly pronounced. Using cuvettes as chemical barriers enabling physical communication  I  placed 5 indicator cells/mL, the inducer populations, into smaller cuvettes that stand in bigger and contained 50, 100, 200 or 300 cells/mL. Under conditions of *total darkness* such pairs were mutually exposed for 48 hours. The hypothesis was that indicator cells, too, grow less the more neighbor cells there are. The bigger inducer populations were in the beginning the less they grew. The indicator populations grew accordingly; the more cells they were surrounded by the less they grew. The suppressing neighbors-effect disappeared when inner cuvettes were shielded by graphite known to shield electromagnetic radiation from GHz to PHz, i.e. to absorb energy from microwaves to light. These are the first results demonstrating non-contact physical *quorum sensing* for cell population density regulation. I assume rules intrinsic to electromagnetic fields interacting with matter and life.

## Introduction

How does a population of cells in a multicellular organism maintain its cell density? Basic understanding is coming from studies with unicellular organisms where cells release chemical signals in dependence of cell density leading to a corresponding regulation of the cell cycle^1–3^. Beside this molecule-based, i.e. chemical *quorum sensing*, there is also (contact-based) physical *quorum sensing* reported in the context of dispersal rates^4^. Further, effects among cells on cell cycle occur also through glass or quartz barriers where chemical signaling is prevented^5–7^. These effects seem to be governed by physical factors, too, most evidently electromagnetic (EM) signals generated by the cells themselves^8,9^, enabling – due to their physical nature – to cross glass or quartz barriers^10^.

In the present study, this seemingly non-chemical cell-to-cell communication was further investigated as its possible biological role was analyzed. The study organism was the freshwater protozoan *Paramecium caudatum*, as already used in previous studies by the present author showing *intraspecific* ^11^ as well as *interspecific* ^12^ effects from one population on the other across chemical barriers.

One possible function of this non-chemical cell communication in *Paramecium* caudatum is assumed to be the regulation of population density. I distinct here the main-hypothesis of density regulation due to a physical signal that can trespass chemical barriers and the sub-hypothesis that these physical signals are of electromagnetic nature.

*Paramecium caudatum* used in this study are maintained in microcosms where they reach an average density of about 300 cells/mL. Even though they receive food (bacteria) *ad libidum* and each cell occupies only about 0.1% of the average volume available for each cell one never finds the cells overshooting that particular density. In the present study  I wanted to find out whether physical signals are used to regulate the cell density in *Paramecium caudatum*. For this,  I placed cells at four different densities, (i.e., 50, 100, 200 and 300 cells per mL) into cuvettes. It is expected that low-density populations would still grow during the course of the experiment whereas populations with higher densities would have a slower growth, and those at carrying capacity would stop growing - or just stay in a balance between cell mortality and division. Yet, what if we place smaller cuvettes containing 5 cells (per mL) into those above-mentioned populations? Would these 5 tester cells act as indicators in that they would grow in dependence on the density of the population they are surrounded by? The hypothesis of a physical (presumably electromagnetic) cell density regulation is based on the conclusion that the bigger the outer population is at the onset of the experiment the lower would not only be their own growth but also the lower would be the growth of those 5 original (indicator) cells placed inside. This was assumed to be so because the presumed endogenous (electromagnetic) signal with which the inducer population regulates its own density trespasses to the inner cuvette, where the tester population adopts its growth decision accordingly, hence indicating that the big inducer population can regulate its density via non-chemical cell-to-cell communication.

The results of this study deliver strong evidence for a physical regulation of population density: across chemical barriers the five inner cells grew less, the more neighbors they had. This indicates that the inducer populations released a signal that regulates cell density. When shielding against presumed electromagnetic signals, the growth decreasing effect from neighboring populations disappeared, supporting the assumption that an endogenous electromagnetic signal was transmitted from the outer inducer population to the inner tester population.

## Results

### Experiment 1: Density-effect

**Figure 1:**
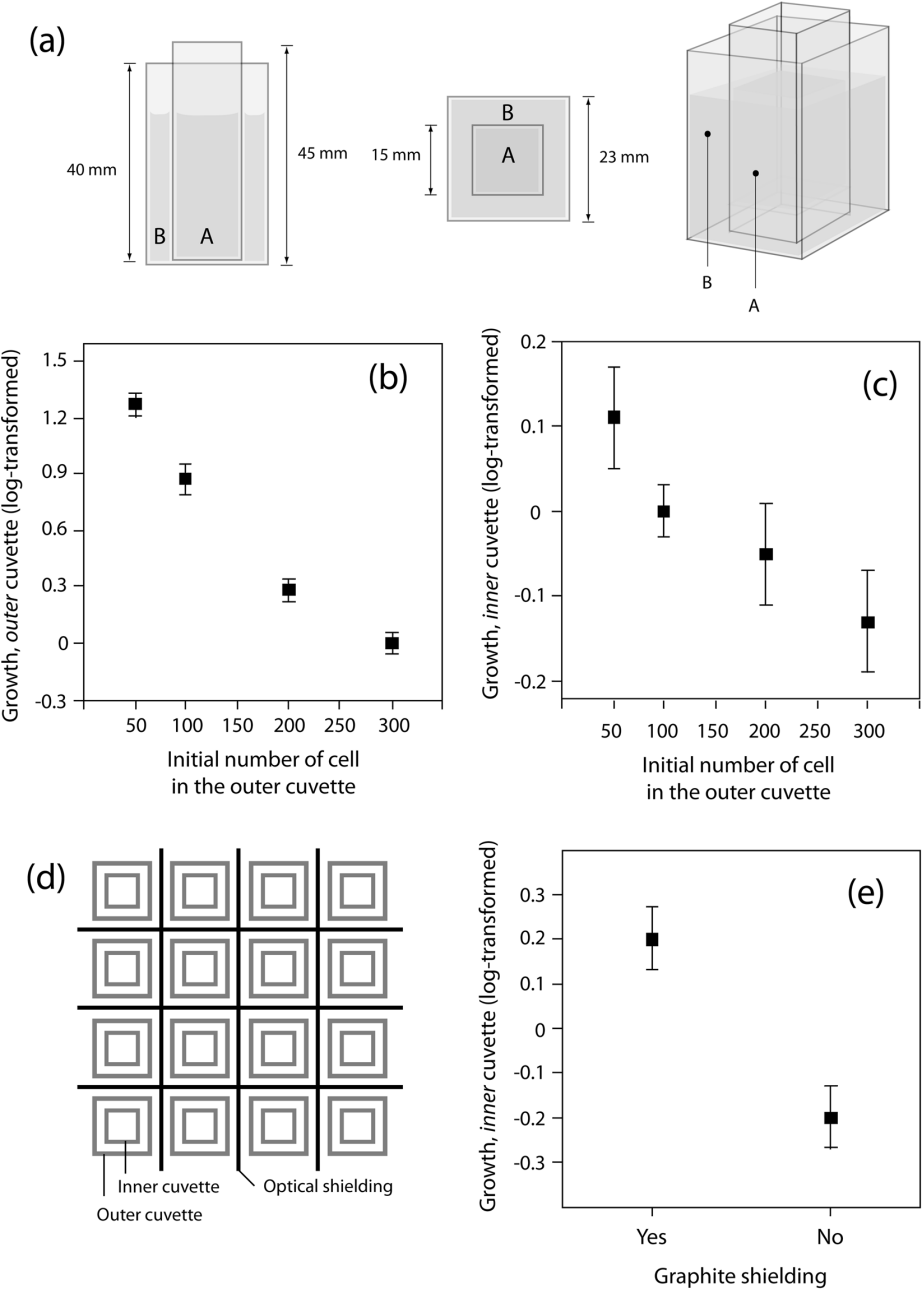
Panel (a) and (d) refer to Material & Method, panel (b), (c) and (e) to Results. (**a**) Visualization of the experimental setup. (**b**) Growth of inducer populations. The x-axis shows the densities of the inducer populations at the beginning of the experiment. The y-axis show log transformed growth rates of these inducer populations. The bigger the inducer populations were at the beginning the less they were growing. (**c**) Growth of tester (indicator) populations. The x-axis shows the densities of the inducer populations at the beginning of the experiment. The y-axis show log transformed growth rates of tester populations. The bigger the inducer populations were at the beginning the less the tester populations were growing. (**d**) Arrangement of units in a grid. All units – small cuvette standing in a big one, i.e. treatment group(s) – have been optically shielded from each other (by black cardboard of 0.33 mm thickness). In a typical experiment, the units are placed in a random design. Note, that volatiles – if existing – would affect the outcome by influencing all units across a microclimate. (**e**) Effect of graphite shielding. Shielded cells grow better than non-shielded tester populations when (both are) having neighbors.

The inducer populations showed a highly significant negative relation between initial population size and cell division rate (ANOVA (linear fit): DF = 1; SS = 16.257; F-ratio = 610.6; p > F = 0.0001****) with the biggest populations (300 cells at origin) performing no more growth (Fig. 1(b)). Cell division rates of tester cells and controls had similar mean values (ANOVA (linear fit): DF = 1; SS = 0.007; F-ratio = 0.148; p > F = 0.702 ns). In either of the above tests there was a highly significant effect from repeating the experiment (Tab 1), but no material effects due to different cuvette material (statistics not shown) which latter allowed to merge the glass and quartz treatment groups which leading then to n = 16 per treatment group.

Regarding the working hypothesis, there was a strong significant negative effect between initial densities of inducer populations and cell division rates of the tester populations (Table 1, Fig. 1(c)). Cases of mortality in the inducer populations were omitted in the above analysis because this adds the variable mortality known for tremendous release of endogenously generated photons^13^. Mortality was assessed in 10 inducer populations with originally 300 cells/mL (8 cases) and 200 cells/mL (2 cases). While tester populations were positively correlated with inducer populations when the latter were growing (ANOVA (linear fit): DF = 1; SS = 0.870; F-ratio = 8.014; p > F = 0.0066**), note that they were negatively correlated when inducer populations displayed mortality i.e., tester populations grew significantly better the higher the mortality rate in the inducer populations (ANOVA (linear fit): DF = 1; SS = 0.490; F-ratio = 9.007; p > F = 0.017*).

**Table 1:** The density-dependent effect of inducer populations on growth in tester populations.

Factor	DF	SS	F-ratio	p < F
Material	1	0.001	0.013	0.908
Experiment	3	4.294	33.272	<0.0001****
Inducer cell density	1	0.326	7.587	0.0083**

Note there were no effects from material (i.e., glass or quartz barrier) but strong effects due to repeating the experiment. (ANOVA; DF = degrees of freedom; SS = sum of squares).

### Experiment 2: Graphite-shielding

Tester populations that were shielded with graphite from inducer populations grew significantly better (ANOVA (linear fit): DF = 1; SS = 0.774; F-ratio = 14.437; p > F = 0.0014**) than those not shielded (Fig. 1(e)).

There was no difference in growth of inducer populations due to exposure to the graphite-layer on the outside of the inner cuvette (statistics not shown).

## Discussion

The results of the present study provide strong evidence for a physical signal organizing the regulation of population density in the unicellular organism *Paramecium caudatum*. This physical signal is most probably of electromagnetic nature since the effect disappeared when shielding tester populations against electromagnetic signals assumed to be emitted by inducer populations. Further, under the shielding conditions, presumed volatiles (i.e., volatile chemical compounds mediating long-range cell-to-cell signaling) could still have induced an effect but, such an effect was absent. The absence of volatiles in the experimental system is extensively discussed elsewhere^11^. Since the results indicate that cells can sense the *quorum*, i.e., the number of cells the population consists in, I suggest here to interpret this study as evidence for a non-contact *physical* (most probably electromagnetic) *quorum sensing*.

Despite the confirmation of the initial hypothesis, the study delivered two unexpected results: (i) The absence of effects from different material types (normal glass or quartz glass) of separating cuvettes was opposed to a previous study with the same cell system^11^. It suggests that during this experiment the inducing signals were in the wavelength-range that could be transmitted through both, glass and quartz cuvettes. Such wavelengths refer to the UV-A range and below. (ii) The effects coming from inducer populations that began to decrease in cell number during the experiment and were inducing a growth increase response in tester populations were an interesting side observation of the experiment and demands an intense investigation on effects of mortality on cell division rates and regulation, respectively.

The major effects described in this study deliver indirect evidence that cells use signals that offshoot from the cellular generation of electromagnetic fields^8,14,15^. Surely, direct evidence is desirable, but as we are not technically able to (i) catch, (ii) store and (iii) release at will electromagnetic waves released by one subpopulation and emitted to another, the here-mentioned method for testing non-chemical cell-to-cell communication is in principal (still) the best; for a detailed discussion about this aspect please refer to Fels^10^. To get more information about the true nature of the signals, the graphite-shielding method is useful but delivers only indirect evidence. For direct evidence, therefore, the use of multimode optical fibers with a transmission window allowing optical communication is planned.

The significance of this study is the support of the assumption that the non-avoidable endogenous generation of electromagnetic fields plays an active role in cell dynamics. This supports the basic hypothesis that the endogenous fields of the cell feedback on cell components some of which having generated these fields^16^. The work is, in addition, mutually supportive to giving credit to works on electrostatic or electromagnetic induction on cell processes being exogenous^17–20^ or endogenous^21–24^.

The results demand to give more attention to the physics of the cell. This may also include to look at effects from physics-based non-invasive therapies and basic research leading to such forms of therapy, respectively^25^. This, however, means that we cannot exclude the possibility that life’s inner organization is also driven by rules intrinsic to electromagnetic fields interacting with matter and life.

## Materials and Methods

### The study organism

*Paramecium caudatum* (Phylum: Ciliata) is a freshwater protozoan of the Eurasian plate and a common research organism in different biological fields such as ecology and cell behaviour^26,27^ or host-parasite interactions^28^. They are large cells with a length of about 250 µm and width of about 50 µm.

In this study, I work with *Paramecia* that are maintained in an incubator with a 24 hrs cycle of 15 hrs artificial light at 23 °C and 9 hrs darkness at 18 °C. Once per week the densities of the cells were assessed with the use of a binocular microscope, glass wells and a hand counter. The cells were thereafter fed with a medium that contains the prokaryote *Serratia sp*. (on which *Paramecia* feed) and dried Lettuce (*Lactuca sativa var*. *capitata*) leaves (on which the prokaryotes feed). Under such conditions, different clones maintain clone specific densities. In this study, the clone K8 was used with an average maximum cell density, i.e., carrying capacity of 300 cells/mL (for more information refer^29^).

### The glass-barrier method

The use of glass-barriers (i.e., cuvettes) goes back to the original works of Alexander Gurwitsch^5^ who employed it to prevent transmission of chemical – but not electromagnetic – signals from one population to another, and was used in many variations ever since^30–32^.

In the present study two populations were separated from each other by using two sizes of cuvettes (vials). Big cuvettes with a base of 23 mm × 23 mm and a height of 40 mm, and small cuvettes with a base of 15 mm × 15 mm and a height of 45 mm the thickness of the walls being 1.5 mm (see Fig. 1(a)). The cuvettes consist either of normal glass (which is a filter for electromagnetic radiation in the optical ultra-violet (UV) spectrum, i.e., UV-B [280–315 nm] and UV-C [100–280 nm]) or quartz glass allowing transmission of the whole UV range; for glass and quartz transmission spectra see elsewhere^11^.

### The separation of two cell populations

In order to separate two cell populations of *Paramecium caudatum*, one is placed in a small cuvette and the other in a bigger cuvette (for a detailed description see^10^). The smaller cuvette is then placed into the big cuvette, leading to a chemical separation of these two populations (Fig. 1(a)). Under the conditions of the experiment each cuvette contains 1 mL of medium (as described above) leading to a height of about 6 mm of medium in the inner and of about 4 mm in the outer cuvette (when containing a small inner cuvette). Such pairs of cuvettes (referred to as “units”) are then randomly placed in a grid (Fig. 1(d)); note that units were separated by black carbon paper disabling light transfer from one unit to the other. The grid itself stands in a lightproof box leading to *total-darkness* conditions during the experiment(s).

### Experiment 1: Density-effect

This experiment tested for a differing effect on tester populations due to differing cell densities in neighbouring inducer populations. At the beginning of an experiment the inducer populations contained either 50, 100, 200 or 300 cells/mL. These densities were obtained by diluting the well-grown clones – reaching population sizes of 300 cells/mL – with medium. The inducer populations were in the outer cuvettes while the tester populations were in the inner cuvettes. The tester populations consisted at the beginning of the experiment always of 5 (individually and randomly picked) cells. A control was added with no inducer population but 1 mL medium in the outer cuvette. The inner and outer cuvettes consisted either both of *normal glass* or *both of quartz* glass. An experimental block contained a random design (Fig. 1(d)) of two replicates in a total of ten treatment groups and was kept for 48 hrs under conditions of total darkness in an incubator at (constant) 26 °C before assessing the growth of the cells. Note, *Paramecia* grow well at room temperature below 30 °C (26). The experiment itself was repeated four times leading to a sample size of n = 8 per treatment group (as there were no effects from separating material glass or quartz found [see results], data could be merged leading to n = 16).

### Experiment 2: Graphite-shielding

Shielding is a commonly used method when looking for electromagnetic effects between organisms or cells^30,33,34^. If the signal in the *Paramecium caudatum* system is electromagnetic, then a thin layer of colloidal graphite around the inner cuvette should prevent electromagnetic signals^35^ coming from the outer inducer population that could induce an effect on the inner tester population. Using purest colloidal graphite in solution (CRAMOLIN® GRAPHIT) a graphite-layer was twice sprayed onto the bottom and up to a height of 15 mm on the outer side of the small cuvettes. Graphite has the capability of strongly decreasing the transmission of electromagnetic signals; it is known to shield electromagnetic field efficiently in the range of radiofrequency/microwave region up to wavelengths in the light spectrum, i.e. from GHz to PHz^35–38^.

The experiment consisted of five cells in the inner (small) cuvettes surrounded by 100 neighbouring cells and separated from each other either with or without an additional graphite-shield. Only quartz cuvettes were in use. An experimental block consisted of five replicates of each treatment group arranged in a random design. Each block was kept for 48 hrs under conditions of total darkness in an incubator at constantly 26 °C before assessing the growth of the cells. The experiment was repeated two times leading to a sample size of n = 10 per treatment group.

### Analysis

All data were log-transformed and analysed with JMP statistics^39^.
